# 2,5-Bis[2-(4-methyl­phen­yl)ethyn­yl]benzyl methacrylate

**DOI:** 10.1107/S1600536811029631

**Published:** 2011-07-30

**Authors:** Zhen-Lin Zhang, Hai-Quan Zhang

**Affiliations:** a State Key Laboratory of Metastable Materials Science and Technology, Yanshan University, Qinhuangdao 066004, People’s Republic of China

## Abstract

In the title bis-tolane derivative, C_29_H_24_O_2_, the central benzene ring forms dihedral angles of 29.12 (9) and 26.46 (9)° with the other two benzene rings. The dihedral angle between two terminal benzene rings is 55.58 (8)°.

## Related literature

For a related structure and the synthesis, see Zhang *et al.* (2010 ▶).
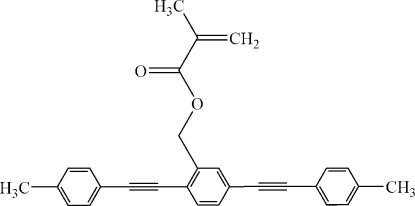


## Experimental

### 

#### Crystal data

C_29_H_24_O_2_
*M* *_r_* = 404.48
Monoclinic, 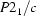

*a* = 13.479 (3) Å
*b* = 10.314 (2) Å
*c* = 18.390 (7) Å
β = 116.06 (2)°
*V* = 2296.7 (11) Å^3^
*Z* = 4
Mo *K*α radiation
μ = 0.07 mm^−1^
*T* = 293 K
0.14 × 0.14 × 0.12 mm

#### Data collection

Rigaku R-AXIS RAPID diffractometer
Absorption correction: multi-scan (*ABSCOR*; Higashi, 1995 ▶) *T*_min_ = 0.990, *T*_max_ = 0.991
21783 measured reflections
5232 independent reflections
3322 reflections with *I* > 2σ(*I*)
*R*_int_ = 0.036

#### Refinement

*R*[*F* ^2^ > 2σ(*F* ^2^)] = 0.052
*wR*(*F* ^2^) = 0.162
*S* = 1.05
5232 reflections
283 parameters
H-atom parameters constrained
Δρ_max_ = 0.22 e Å^−3^
Δρ_min_ = −0.16 e Å^−3^

### 

Data collection: *RAPID-AUTO* (Rigaku, 1998 ▶); cell refinement: *RAPID-AUTO*; data reduction: *CrystalStructure* (Rigaku/MSC, 2002 ▶); program(s) used to solve structure: *SHELXS97* (Sheldrick, 2008 ▶); program(s) used to refine structure: *SHELXL97* (Sheldrick, 2008 ▶); molecular graphics: *PLATON* (Spek, 2009 ▶); software used to prepare material for publication: *SHELXL97*.

## Supplementary Material

Crystal structure: contains datablock(s) global, I. DOI: 10.1107/S1600536811029631/aa2017sup1.cif

Structure factors: contains datablock(s) I. DOI: 10.1107/S1600536811029631/aa2017Isup2.hkl

Supplementary material file. DOI: 10.1107/S1600536811029631/aa2017Isup3.cml

Additional supplementary materials:  crystallographic information; 3D view; checkCIF report

## Figures and Tables

**Fig. 1.**
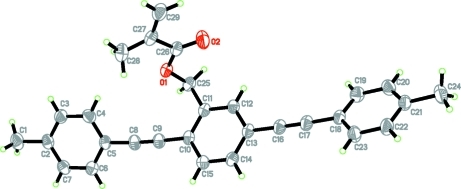
The crystal structure of the title compound, with the atom numbering. Displacement ellipsoids of non-H atoms are drawn at the 30% probalility level.

## supplementary crystallographic information

### Comment

High birefringence liquid crystals are useful not only in conventional display devices such as STNLCDs, but also in scattering-type PDLCDs as a reflective LCD, and in spatial light modulators.They are also of interest as componens of LCDs; for example, compensation films for improving the viewing angle, reflectors and polarizers. The bistolane derivatives is very important kind of high birefringence material. We have reported the similar synthesis of the compound in previous paper (Zhang *et al.* 2010). Herein we present the crystal structure of the title compound (see Fig. 1). All bond lengths and angles are in the normal ranges. The three benzene rings of the title compound are not coplanar, and the dihedral angles between the side-benzene rings and the central benzene ring are 29.12 (9)° and 26.46 (9)°, respectively. The dihedral angle between two terminal benzene rings is 55.58 (8)°. The crystal packing is stabilized by Van der Waals' force.

### Experimental

The title compound was prepared according to the literature (Zhang *et al.*, 2010). Single crystals suitable for X-ray diffraction were prepared by slow evaporation of a dichloromethane solution at room temperature.

### Refinement

H-atoms were placed in calculated positions and were included in the refinement in the riding model with C—H distances 0.93 Å for aromatic C—H and =CH_2_, 0.97 Å for —CH_2_— and 0.96 Å for —CH_3_. *U*_iso_(H) = 1.5 *U*_eq_(C) for methyl H atoms and 1.2 *U*_eq_(C) for the rest H atoms.

### Crystal data

**Table:**

C_29_H_24_O_2_	*F*(000) = 856
*M**_r_* = 404.48	*D*_x_ = 1.170 Mg m^−^^3^
Monoclinic, *P*2_1_/*c*	Mo *K*α radiation, λ = 0.71073 Å
Hall symbol: -P 2ybc	Cell parameters from 5232 reflections
*a* = 13.479 (3) Å	θ = 3.0–27.5°
*b* = 10.314 (2) Å	µ = 0.07 mm^−^^1^
*c* = 18.390 (7) Å	*T* = 293 K
β = 116.06 (2)°	Block, colourless
*V* = 2296.7 (11) Å^3^	0.14 × 0.14 × 0.12 mm
*Z* = 4	

### Data collection

**Table:**

Rigaku R-AXIS RAPID diffractometer	5232 independent reflections
Radiation source: fine-focus sealed tube	3322 reflections with *I* > 2σ(*I*)
graphite	*R*_int_ = 0.036
ω scans	θ_max_ = 27.5°, θ_min_ = 3.0°
Absorption correction: multi-scan (*ABSCOR*; Higashi, 1995)	*h* = −17→17
*T*_min_ = 0.990, *T*_max_ = 0.991	*k* = −13→13
21783 measured reflections	*l* = −23→23

### Refinement

**Table:**

Refinement on *F*^2^	Primary atom site location: structure-invariant direct methods
Least-squares matrix: full	Secondary atom site location: difference Fourier map
*R*[*F*^2^ > 2σ(*F*^2^)] = 0.052	Hydrogen site location: inferred from neighbouring sites
*wR*(*F*^2^) = 0.162	H-atom parameters constrained
*S* = 1.05	*w* = 1/[σ^2^(*F*_o_^2^) + (0.090*P*)^2^ + 0.0263*P*] where *P* = (*F*_o_^2^ + 2*F*_c_^2^)/3
5232 reflections	(Δ/σ)_max_ = 0.026
283 parameters	Δρ_max_ = 0.22 e Å^−^^3^
0 restraints	Δρ_min_ = −0.16 e Å^−^^3^

### Special details

**Table:**

Experimental. (See detailed section in the paper)
Geometry. All s.u.'s (except the s.u. in the dihedral angle between two l.s. planes) are estimated using the full covariance matrix. The cell s.u.'s are taken into account individually in the estimation of s.u.'s in distances, angles and torsion angles; correlations between s.u.'s in cell parameters are only used when they are defined by crystal symmetry. An approximate (isotropic) treatment of cell s.u.'s is used for estimating s.u.'s involving l.s. planes.
Refinement. Refinement of *F*^2^ against ALL reflections. The weighted *R*-factor *wR* and goodness of fit *S* are based on *F*^2^, conventional *R*-factors *R* are based on *F*, with *F* set to zero for negative *F*^2^. The threshold expression of *F*^2^ > σ(*F*^2^) is used only for calculating *R*-factors(gt) *etc*. and is not relevant to the choice of reflections for refinement. *R*-factors based on *F*^2^ are statistically about twice as large as those based on *F*, and *R*- factors based on ALL data will be even larger.

### Fractional atomic coordinates and isotropic or equivalent isotropic displacement parameters (Å^2^)

**Table:**

	*x*	*y*	*z*	*U*_iso_*/*U*_eq_	
O1	0.46919 (9)	0.83960 (11)	0.94444 (6)	0.0577 (3)	
O2	0.31533 (11)	0.86679 (15)	0.95953 (9)	0.0855 (4)	
C1	1.02239 (16)	0.9345 (3)	0.88082 (12)	0.0898 (7)	
H1A	1.0037	0.9592	0.8260	0.135*	
H1B	1.0873	0.8811	0.9011	0.135*	
H1C	1.0364	1.0109	0.9137	0.135*	
C2	0.92826 (13)	0.8602 (2)	0.88343 (9)	0.0622 (5)	
C3	0.85824 (15)	0.9179 (2)	0.90984 (10)	0.0683 (5)	
H3	0.8717	1.0029	0.9285	0.082*	
C4	0.76866 (14)	0.8535 (2)	0.90948 (11)	0.0657 (5)	
H4	0.7228	0.8952	0.9278	0.079*	
C5	0.74624 (12)	0.72585 (18)	0.88170 (9)	0.0549 (4)	
C6	0.81733 (13)	0.66578 (19)	0.85598 (9)	0.0584 (4)	
H6	0.8044	0.5806	0.8376	0.070*	
C7	0.90735 (13)	0.7320 (2)	0.85748 (10)	0.0620 (5)	
H7	0.9549	0.6900	0.8408	0.074*	
C8	0.65017 (13)	0.66127 (19)	0.87854 (10)	0.0607 (4)	
C9	0.56739 (13)	0.61534 (18)	0.87514 (9)	0.0579 (4)	
C10	0.46640 (12)	0.56551 (16)	0.87212 (9)	0.0503 (4)	
C11	0.42631 (11)	0.61130 (15)	0.92603 (8)	0.0487 (4)	
C12	0.32830 (12)	0.56368 (16)	0.92154 (9)	0.0518 (4)	
H12	0.3025	0.5933	0.9578	0.062*	
C13	0.26695 (12)	0.47142 (16)	0.86327 (9)	0.0524 (4)	
C14	0.30682 (13)	0.42763 (17)	0.80977 (9)	0.0559 (4)	
H14	0.2663	0.3673	0.7703	0.067*	
C15	0.40513 (13)	0.47212 (17)	0.81438 (9)	0.0561 (4)	
H15	0.4315	0.4401	0.7789	0.067*	
C16	0.16510 (13)	0.42429 (17)	0.85986 (10)	0.0588 (4)	
C17	0.08020 (13)	0.38608 (18)	0.85793 (10)	0.0609 (4)	
C18	−0.02216 (13)	0.34409 (17)	0.85662 (9)	0.0545 (4)	
C19	−0.03831 (14)	0.35079 (19)	0.92580 (10)	0.0648 (5)	
H19	0.0188	0.3789	0.9741	0.078*	
C20	−0.13764 (15)	0.3163 (2)	0.92386 (10)	0.0670 (5)	
H20	−0.1464	0.3212	0.9712	0.080*	
C21	−0.22507 (13)	0.27457 (17)	0.85347 (10)	0.0587 (4)	
C22	−0.20792 (14)	0.2662 (2)	0.78517 (10)	0.0677 (5)	
H22	−0.2651	0.2375	0.7371	0.081*	
C23	−0.10906 (14)	0.2989 (2)	0.78607 (10)	0.0686 (5)	
H23	−0.0998	0.2908	0.7391	0.082*	
C24	−0.33470 (16)	0.2402 (2)	0.85113 (14)	0.0862 (6)	
H24A	−0.3242	0.1757	0.8915	0.129*	
H24B	−0.3827	0.2066	0.7986	0.129*	
H24C	−0.3673	0.3162	0.8617	0.129*	
C25	0.48771 (13)	0.71594 (16)	0.98580 (9)	0.0577 (4)	
H25A	0.4623	0.7200	1.0276	0.069*	
H25B	0.5661	0.6964	1.0114	0.069*	
C26	0.37595 (13)	0.90213 (17)	0.93213 (9)	0.0570 (4)	
C27	0.35602 (14)	1.01911 (17)	0.87936 (9)	0.0602 (4)	
C28	0.42569 (17)	1.0441 (2)	0.84353 (11)	0.0796 (6)	
H28A	0.4046	1.1243	0.8141	0.119*	
H28B	0.4202	0.9749	0.8071	0.119*	
H28C	0.5004	1.0505	0.8847	0.119*	
C29	0.26432 (16)	1.0942 (2)	0.86929 (13)	0.0811 (6)	
H29A	0.2461	1.1673	0.8364	0.097*	
H29B	0.2216	1.0709	0.8955	0.097*	

### Atomic displacement parameters (Å^2^)

**Table:**

	*U*^11^	*U*^22^	*U*^33^	*U*^12^	*U*^13^	*U*^23^
O1	0.0557 (7)	0.0505 (7)	0.0649 (7)	−0.0080 (5)	0.0246 (5)	0.0027 (5)
O2	0.0758 (9)	0.0834 (10)	0.1147 (11)	0.0109 (7)	0.0579 (8)	0.0339 (8)
C1	0.0726 (13)	0.118 (2)	0.0835 (13)	−0.0401 (13)	0.0388 (10)	−0.0140 (12)
C2	0.0500 (9)	0.0826 (14)	0.0505 (9)	−0.0163 (9)	0.0190 (7)	−0.0017 (8)
C3	0.0696 (11)	0.0693 (13)	0.0667 (11)	−0.0188 (9)	0.0305 (9)	−0.0104 (8)
C4	0.0583 (10)	0.0750 (13)	0.0695 (10)	−0.0031 (9)	0.0334 (8)	−0.0063 (9)
C5	0.0429 (8)	0.0647 (11)	0.0548 (9)	−0.0044 (7)	0.0193 (6)	0.0062 (7)
C6	0.0491 (9)	0.0597 (11)	0.0597 (9)	−0.0011 (8)	0.0178 (7)	0.0045 (7)
C7	0.0459 (8)	0.0798 (13)	0.0599 (9)	0.0026 (8)	0.0231 (7)	0.0046 (8)
C8	0.0467 (9)	0.0706 (12)	0.0620 (10)	−0.0033 (8)	0.0211 (7)	0.0084 (8)
C9	0.0472 (9)	0.0640 (11)	0.0593 (9)	−0.0022 (8)	0.0206 (7)	0.0073 (7)
C10	0.0401 (8)	0.0523 (9)	0.0531 (8)	−0.0007 (6)	0.0154 (6)	0.0107 (7)
C11	0.0421 (8)	0.0456 (9)	0.0494 (8)	−0.0001 (6)	0.0119 (6)	0.0084 (6)
C12	0.0463 (8)	0.0525 (10)	0.0553 (8)	0.0007 (7)	0.0212 (6)	0.0069 (7)
C13	0.0424 (8)	0.0476 (9)	0.0612 (9)	−0.0024 (7)	0.0171 (6)	0.0080 (7)
C14	0.0509 (9)	0.0536 (10)	0.0554 (9)	−0.0065 (7)	0.0161 (7)	0.0009 (7)
C15	0.0523 (9)	0.0583 (10)	0.0573 (9)	−0.0020 (8)	0.0237 (7)	0.0023 (7)
C16	0.0479 (9)	0.0551 (10)	0.0690 (10)	−0.0053 (8)	0.0216 (7)	0.0021 (8)
C17	0.0507 (9)	0.0574 (11)	0.0724 (11)	−0.0068 (8)	0.0249 (8)	0.0017 (8)
C18	0.0501 (9)	0.0485 (9)	0.0641 (9)	−0.0054 (7)	0.0245 (7)	0.0030 (7)
C19	0.0566 (10)	0.0726 (13)	0.0562 (9)	−0.0077 (8)	0.0166 (7)	−0.0021 (8)
C20	0.0693 (11)	0.0791 (14)	0.0604 (10)	−0.0067 (9)	0.0358 (8)	−0.0004 (8)
C21	0.0548 (9)	0.0561 (11)	0.0700 (10)	−0.0057 (8)	0.0320 (8)	0.0037 (8)
C22	0.0567 (10)	0.0817 (14)	0.0618 (10)	−0.0236 (9)	0.0233 (8)	−0.0106 (9)
C23	0.0653 (11)	0.0865 (14)	0.0605 (10)	−0.0212 (10)	0.0335 (8)	−0.0097 (9)
C24	0.0667 (12)	0.1030 (18)	0.1036 (15)	−0.0148 (12)	0.0509 (11)	−0.0018 (12)
C25	0.0532 (9)	0.0528 (10)	0.0553 (9)	−0.0048 (7)	0.0131 (7)	0.0041 (7)
C26	0.0530 (9)	0.0551 (11)	0.0582 (9)	−0.0084 (8)	0.0201 (7)	0.0009 (7)
C27	0.0602 (10)	0.0513 (10)	0.0579 (9)	−0.0139 (8)	0.0157 (7)	0.0005 (7)
C28	0.0817 (13)	0.0797 (15)	0.0774 (12)	−0.0135 (11)	0.0350 (10)	0.0150 (10)
C29	0.0765 (13)	0.0589 (13)	0.1095 (15)	0.0069 (10)	0.0422 (11)	0.0221 (11)

### Geometric parameters (Å, °)

**Table:**

O1—C26	1.340 (2)	C14—H14	0.9300
O1—C25	1.449 (2)	C15—H15	0.9300
O2—C26	1.1900 (19)	C16—C17	1.196 (2)
C1—C2	1.501 (2)	C17—C18	1.436 (2)
C1—H1A	0.9600	C18—C19	1.383 (2)
C1—H1B	0.9600	C18—C23	1.392 (2)
C1—H1C	0.9600	C19—C20	1.371 (2)
C2—C3	1.372 (2)	C19—H19	0.9300
C2—C7	1.392 (3)	C20—C21	1.382 (2)
C3—C4	1.375 (2)	C20—H20	0.9300
C3—H3	0.9300	C21—C22	1.376 (2)
C4—C5	1.397 (3)	C21—C24	1.502 (2)
C4—H4	0.9300	C22—C23	1.368 (2)
C5—C6	1.387 (2)	C22—H22	0.9300
C5—C8	1.434 (2)	C23—H23	0.9300
C6—C7	1.382 (2)	C24—H24A	0.9600
C6—H6	0.9300	C24—H24B	0.9600
C7—H7	0.9300	C24—H24C	0.9600
C8—C9	1.188 (2)	C25—H25A	0.9700
C9—C10	1.433 (2)	C25—H25B	0.9700
C10—C15	1.402 (2)	C26—C27	1.497 (2)
C10—C11	1.403 (2)	C27—C28	1.388 (3)
C11—C12	1.377 (2)	C27—C29	1.400 (3)
C11—C25	1.503 (2)	C28—H28A	0.9600
C12—C13	1.400 (2)	C28—H28B	0.9600
C12—H12	0.9300	C28—H28C	0.9600
C13—C14	1.388 (2)	C29—H29A	0.9300
C13—C16	1.431 (2)	C29—H29B	0.9300
C14—C15	1.369 (2)		
			
C26—O1—C25	116.40 (13)	C16—C17—C18	178.2 (2)
C2—C1—H1A	109.5	C19—C18—C23	117.78 (15)
C2—C1—H1B	109.5	C19—C18—C17	120.51 (15)
H1A—C1—H1B	109.5	C23—C18—C17	121.69 (15)
C2—C1—H1C	109.5	C20—C19—C18	120.65 (15)
H1A—C1—H1C	109.5	C20—C19—H19	119.7
H1B—C1—H1C	109.5	C18—C19—H19	119.7
C3—C2—C7	117.78 (16)	C19—C20—C21	121.77 (15)
C3—C2—C1	120.8 (2)	C19—C20—H20	119.1
C7—C2—C1	121.40 (18)	C21—C20—H20	119.1
C2—C3—C4	121.86 (19)	C22—C21—C20	117.29 (16)
C2—C3—H3	119.1	C22—C21—C24	121.11 (16)
C4—C3—H3	119.1	C20—C21—C24	121.60 (16)
C3—C4—C5	120.34 (17)	C23—C22—C21	121.79 (15)
C3—C4—H4	119.8	C23—C22—H22	119.1
C5—C4—H4	119.8	C21—C22—H22	119.1
C6—C5—C4	118.36 (16)	C22—C23—C18	120.68 (15)
C6—C5—C8	121.69 (17)	C22—C23—H23	119.7
C4—C5—C8	119.93 (16)	C18—C23—H23	119.7
C7—C6—C5	120.30 (18)	C21—C24—H24A	109.5
C7—C6—H6	119.9	C21—C24—H24B	109.5
C5—C6—H6	119.9	H24A—C24—H24B	109.5
C6—C7—C2	121.34 (17)	C21—C24—H24C	109.5
C6—C7—H7	119.3	H24A—C24—H24C	109.5
C2—C7—H7	119.3	H24B—C24—H24C	109.5
C9—C8—C5	175.8 (2)	O1—C25—C11	109.61 (12)
C8—C9—C10	177.4 (2)	O1—C25—H25A	109.7
C15—C10—C11	118.99 (14)	C11—C25—H25A	109.7
C15—C10—C9	120.60 (14)	O1—C25—H25B	109.7
C11—C10—C9	120.40 (15)	C11—C25—H25B	109.7
C12—C11—C10	119.64 (14)	H25A—C25—H25B	108.2
C12—C11—C25	120.35 (14)	O2—C26—O1	123.25 (16)
C10—C11—C25	119.95 (14)	O2—C26—C27	124.08 (17)
C11—C12—C13	121.12 (14)	O1—C26—C27	112.66 (14)
C11—C12—H12	119.4	C28—C27—C29	125.20 (18)
C13—C12—H12	119.4	C28—C27—C26	119.44 (17)
C14—C13—C12	118.78 (14)	C29—C27—C26	115.34 (16)
C14—C13—C16	121.41 (15)	C27—C28—H28A	109.5
C12—C13—C16	119.81 (15)	C27—C28—H28B	109.5
C15—C14—C13	120.85 (15)	H28A—C28—H28B	109.5
C15—C14—H14	119.6	C27—C28—H28C	109.5
C13—C14—H14	119.6	H28A—C28—H28C	109.5
C14—C15—C10	120.59 (15)	H28B—C28—H28C	109.5
C14—C15—H15	119.7	C27—C29—H29A	120.0
C10—C15—H15	119.7	C27—C29—H29B	120.0
C17—C16—C13	179.05 (19)	H29A—C29—H29B	120.0
			
C7—C2—C3—C4	−1.3 (3)	C11—C10—C15—C14	1.0 (2)
C1—C2—C3—C4	177.14 (16)	C9—C10—C15—C14	−177.85 (14)
C2—C3—C4—C5	−0.1 (3)	C23—C18—C19—C20	−1.3 (3)
C3—C4—C5—C6	1.0 (2)	C17—C18—C19—C20	177.03 (17)
C3—C4—C5—C8	−177.52 (15)	C18—C19—C20—C21	−0.3 (3)
C4—C5—C6—C7	−0.5 (2)	C19—C20—C21—C22	1.3 (3)
C8—C5—C6—C7	178.01 (13)	C19—C20—C21—C24	−178.41 (19)
C5—C6—C7—C2	−0.9 (2)	C20—C21—C22—C23	−0.7 (3)
C3—C2—C7—C6	1.9 (2)	C24—C21—C22—C23	179.1 (2)
C1—C2—C7—C6	−176.61 (15)	C21—C22—C23—C18	−1.0 (3)
C15—C10—C11—C12	0.2 (2)	C19—C18—C23—C22	2.0 (3)
C9—C10—C11—C12	179.08 (14)	C17—C18—C23—C22	−176.36 (18)
C15—C10—C11—C25	−177.08 (14)	C26—O1—C25—C11	83.10 (17)
C9—C10—C11—C25	1.8 (2)	C12—C11—C25—O1	−102.30 (16)
C10—C11—C12—C13	−0.9 (2)	C10—C11—C25—O1	75.01 (18)
C25—C11—C12—C13	176.44 (14)	C25—O1—C26—O2	7.0 (2)
C11—C12—C13—C14	0.3 (2)	C25—O1—C26—C27	−172.09 (13)
C11—C12—C13—C16	−179.67 (14)	O2—C26—C27—C28	−173.18 (18)
C12—C13—C14—C15	1.0 (2)	O1—C26—C27—C28	5.9 (2)
C16—C13—C14—C15	−179.08 (15)	O2—C26—C27—C29	5.6 (3)
C13—C14—C15—C10	−1.6 (2)	O1—C26—C27—C29	−175.33 (15)
